# The dynamic evolution of circulating tumor cells during glecirasib treatment predicts survival and resistance in gastrointestinal tumors with KRAS^*G12C*^ mutation

**DOI:** 10.1007/s13577-026-01405-0

**Published:** 2026-07-01

**Authors:** Lei Jiang, Yiming Luo, Huan Liang, Xujiao Feng, Yan Huang, Yanyan Li, Zhenghang Wang, Ting Xu, Heng Zou, Zhi Li, Lin Shen, Yang Chen, Weihu Wang, Jian Li

**Affiliations:** 1 Department of Gastrointestinal Oncology, Key Laboratory of Carcinogenesis and Translational Research (Ministry of Education), Peking University Cancer Hospital and Institute, Beijing, China; 2 Department of Radiation Oncology, Key Laboratory of Carcinogenesis and Translational Research, Ministry of Education/Beijing), Peking University Cancer Hospital and Institute, Haidian District, Fucheng Road 52, Beijing, China; 3 Department of Medical Affairs, Cellomics (Shen Zhen) Limited, Shenzhen, China

**Keywords:** JAB-21822, KRAS G12C, Glecirasib, CTCs, Gastrointestinal tumors

## Abstract

The Kirsten rat sarcoma viral oncogene homolog (KRAS) is one of the most frequently mutated oncogenes and is associated with poor prognosis. Long considered an undruggable target, KRAS has recently become actionable with the development of direct inhibitors, particularly against the G12C mutation. Our group previously reported promising efficacy and safety results for glecirasib (JAB-21822), a novel KRAS^*G12C*^ inhibitor, in phase I/II trials involving solid tumors harboring KRAS^*G12C*^ mutations (ClinicalTrials.gov NCT05009329, NCT05194995). Nevertheless, primary (5.61%) and acquired (9.64%) resistance were observed. This study analyzed 18 patients with advanced solid tumors harboring the KRAS^*G12C*^ mutation from the JAB-21822 cohort. Longitudinal blood samples (*N* = 45) were collected at baseline, during partial response or stable disease, and at disease progression. Circulating tumor cells (CTCs) were isolated via a microfluidics platform (CTC100, Cellomics) and categorized into epithelial (E-CTCs), mesenchymal (M-CTCs), and epithelial/mesenchymal mixed (E/M-CTCs) subtypes. At progression, the proportion of E-CTCs showed a decreased trend, while that of E/M-CTCs increased significantly (*p* = 0.03). In long-term responders, the inflection points of declining M-CTC levels correlated with clinical progression and were consistent with radiographic outcomes. Baseline CTC counts > 1 were associated with shorter progression-free survival (PFS; *p* = 0.046). E-CTC ≤ 1 correlated with longer PFS (*p* = 0.025), and M-CTC ≤ 1 with longer overall survival (OS; *p* = 0.033). E/M-CTC ≤ 1 showed a trend toward improved OS (*p* = 0.086). In addition, patients with > 1 CTC who received local radiotherapy for progressive lesions after glecirasib targeted therapy had significantly prolonged PFS and OS compared to those who did not (*p* < 0.05).

**Graphical Abstract:**

**Figure:**
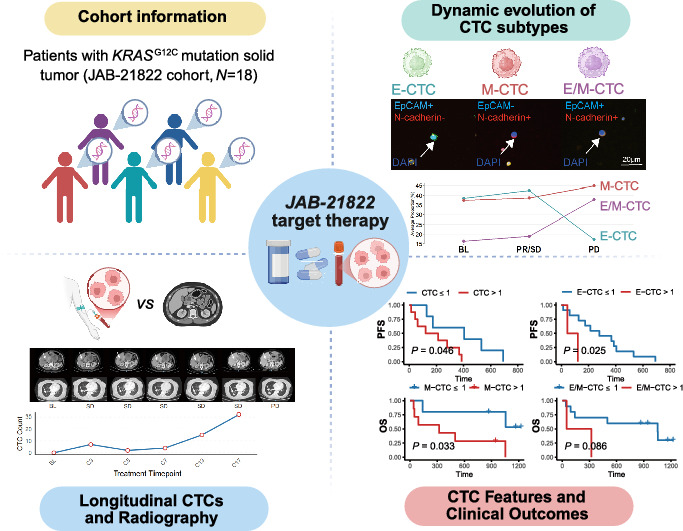

**Supplementary Information:**

The online version contains supplementary material available at 10.1007/s13577-026-01405-0.

## Introduction

Kirsten rat sarcoma viral oncogene homolog (KRAS), a member of the RAS GTPase family, is the most frequently mutated oncogene in human cancers [1]. It functions as a pivotal oncogenic driver, regulating cell proliferation, survival, and differentiation primarily through the MAPK signaling pathway [2]. Among these mutations, the glycine-to-cysteine substitution at codon 12 of the *KRAS* oncogene (KRAS^*G12C*^) constitutes a significant subset across multiple solid tumors, including approximately 3–5% of colorectal cancers (CRC), 11–16% of non-small cell lung cancers (NSCLC), 1–3% of pancreatic ductal adenocarcinoma (PDAC), 0.6% of gastric cancer (GC), and notable subsets of cholangiocarcinoma (CCA) [3–5]. Patients harboring *KRAS*^*G12C*^ mutation face a particularly poor prognosis, characterized by shorter progression-free survival (PFS) and overall survival (OS) as compared to those with other *KRAS* variants or wild-type disease^1^. In metastatic CRC, the KRAS^*G12C*^ was linked to reduced median OS (18.2 vs. 19.1 months) and real-world PFS (7.1 vs. 8.9 months) [6]. For decades, the direct targeting of this “undruggable” mutation remained a formidable therapeutic challenge, leaving patients with limited effective treatment options beyond conventional chemotherapy [1].

The development of allele-specific covalent inhibitors, such as sotorasib and adagrasib that trap *KRAS*^*G12C*^ in its inactive GDP-bound state, has revolutionized the therapeutic landscape for *KRAS*^*G12C*^ mutant cancers, demonstrating clinically meaningful efficacy and leading to regulatory approvals [1, 7, 8]. Our group led a phase I/II clinical trial (ClinicalTrials.gov NCT05009329 and NCT05194995) to evaluate the efficacy and safety of glecirasib (JAB-21822), a novel KRAS^*G12C*^ inhibitor, as monotherapy or in combination with the EGFR inhibitor cetuximab for patients with advanced solid tumors [4, 9]. Among the 44 patients who received monotherapy, the objective response rate (ORR) was 23%, the disease control rate (DCR) reached 86%, the median progression-free survival (mPFS) was 5.6 months, and the median overall survival (mOS) was 16.0 months, with 85% of patients experiencing tumor shrinkage. In the 46 patients treated with the combination of glecirasib and cetuximab, efficacy was significantly enhanced: ORR increased to 50%, DCR was 87%, mPFS extended to 6.9 months, and mOS reached 19.3 months [9]. However, based on the treatment response data from our group's clinical study of JAB-21822 cohort, early radiographic assessment revealed primary and acquired resistance rates of 5.61% (5/83) and 9.64% (8/83), respectively [4, 9]. This rapid emergence of resistance underscores a critical unmet need to understand and monitor the dynamic evolution of tumors under therapeutic pressure.

Circulating tumor cells (CTCs), as viable mediators of metastasis, offer a unique “liquid biopsy” modality to directly interrogate tumor biology in vivo [10, 11]. They serve as dynamic reservoirs of cellular phenotypic plasticity, such as epithelial-to-mesenchymal transition (EMT) and chromosomal instability [12], features intimately linked to metastatic competence and therapy resistance. In gastric cancer, for instance, the heterogeneity in CTCs size and phenotype has been conclusively linked to prognosis and resistance to chemo-targeted therapies [13]. A key advantage of CTCs analysis is its potential to detect molecular and cellular changes indicative of treatment failure prior to radiographic progression on standard imaging (e.g., CT scans) [14]. Therefore, this study utilized a prospective longitudinal cohort of 18 patients with KRAS^*G12C*^ mutation solid tumors (NCT05009329, NCT05194995) treated with glecirasib (JAB-21822 cohort). It aimed to dynamically characterize the evolution of CTCs subtypes and evaluate their prognostic value.

## Materials and methods

### Specimen collection

This study comprised 45 peripheral blood samples from 18 patients with KRAS^*G12C*^ mutation gastrointestinal tumor. These samples were collected longitudinally between Sep. 2022 and Mar. 2024, and the patients received glecirasib therapy. Treatment efficacy was assessed according to the response evaluation criteria in solid tumors (RECIST 1.1) criteria. The post-treated samples were further categorized into partial response (PR), stable disease (SD), and progressive disease (PD) samples. All samples were obtained from the Department of Gastrointestinal Oncology at Peking University Cancer Hospital. All participants signed informed consents.

### Isolation of CTCs

CTCs were isolated from 4 mL of whole peripheral blood using a microfluidics platform (CTC100, Cellomics) [15, 16]. The system employs a microfluidic inertial sorting chip with a curved microchannel that utilizes the balance between net lift force and dean flow to separate cells based on size, shape, and rigidity. Peripheral blood mononuclear cells (PBMCs), which contain CTCs, were first obtained from the blood sample via density gradient centrifugation. The PBMC sample and PBS buffer were simultaneously infused into the microfluidic chip using pumps connected to the sample inlet. Sterile tubes were attached to the CTC outlet and waste outlet. As the sample passed through the chip, CTCs were isolated based on the abovementioned physical properties and finally collected from the CTC outlet.

### Immunofluorescence staining

The CTCs collecting tube was centrifuged at 500 × g for 10 min at room temperature (RT). The supernatant was removed, and the cell pellet was gently resuspended in PBS buffer, attached by cytocentrifugation. The supernatant was removed, 200 µL of fixation buffer (4% paraformaldehyde solution) was added to the collected cells, and the buffer solution was incubated for 5 min. The cells were washed three times with PBS (5 min for each wash). The fixed cells were then permeabilized by 0.1% Triton X-100 dissolved in PBS buffer for 10 min and rinsed three times with PBS (5 min for each wash). The cells were then stained with PE-labelled anti-CD45 (1:100, Invitrogen), FITC-labelled anti-EpCAM (1:100, Novus), and AF647-labelled anti-N-cadherin (1:100, Novus) by incubating overnight at 4 °C. After incubation, the cells were rinsed with PBS buffer three times and stained with DAPI for another 5 min. Finally, the collected CTCs were observed under a fluorescence microscope (Olympus IX73) [16]. CTCs showed DAPI^+^/CD45^−^, based on the expression of EpCAM (epithelial marker) and N-cadherin (mesenchymal marker), three CTCs subtypes were defined, EpCAM^+^/N-cadherin^−^ epithelial CTCs (E-CTCs), EpCAM^−^/N-cadherin^+^ mesenchymal CTCs (M-CTCs) and EpCAM^+^/N-cadherin^+^ mixed CTCs (E/M-CTCs). WBCs showed DAPI^+^/CD45^+^/EpCAM^−^/N-cadherin^−^. The size of CTCs was much larger than WBCs.

### Statistical analysis

Statistical analyses were performed using *R* (4.2.2). The data are presented as mean ± standard deviation (mean ± SD), median with interquartile range (IQR), or frequencies and percentages, depending on the data type and distribution. Comparisons between two groups were conducted using the Student’s *t* test or the Mann–Whitney *U* test. Survival curves were generated using the Kaplan–Meier method, and differences between groups were assessed using the Log-rank test. Firth’s penalized multivariate Cox proportional hazards regression analyses were performed to evaluate the independent prognostic value of baseline CTC parameters, using R (version 4.2.2) with the logistf package. The results are expressed as hazard ratios (HRs) with 95% confidence intervals (CIs). All statistical tests were two-sided, and a *P* value of less than 0.05 was considered statistically significant.

## Results

### Study design and clinicopathological characteristics of patients in the JAB-21822 cohort

**Fig. 1:**
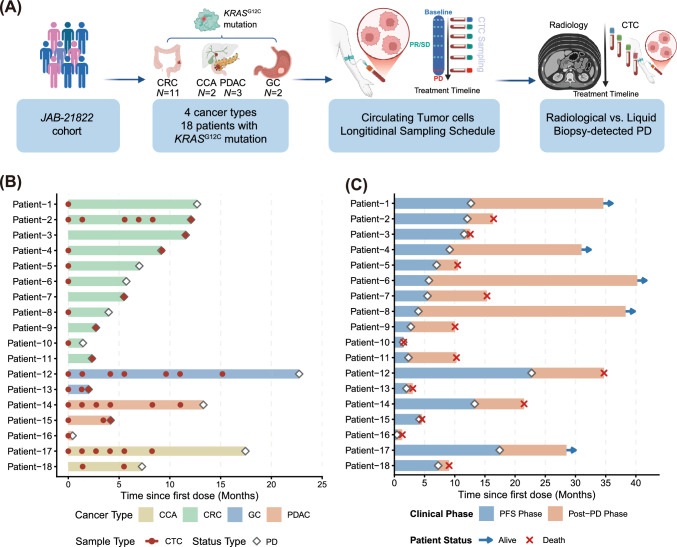
Study design and patient characteristics. **A** Schematic of the longitudinal sample collection protocol. Peripheral blood for CTC analysis was collected at baseline (BL), during radiographic response (PR/SD), and at confirmed disease progression (PD), in parallel with serial CT imaging. **B** Patient-level timeline illustrating the schedule of CTC blood draws aligned with treatment cycles for all 18 patients in the JAB-21822 cohort. **C** Swimmer plot summarizing progression-free survival (PFS) and overall survival (OS) events for the entire cohort

This study utilized data from the previously reported JAB-21822 clinical trial cohort (JAB-21822 cohort) by our group [9], which enrolled 18 patients with advanced solid tumors harboring KRAS^*G12C*^ mutation, all of whom were treated with glecirasib (JAB-21822). The cohort included 11 (61.1%) CRC, 3 (16.7%) PDAC, 2 (11.1%) GC, and 2 (11.1%) CCA patients. Baseline demographic and clinicopathological characteristics of the entire cohort are provided in (Table 1). The study design and sample collection scheme are illustrated (Fig. 1A). Longitudinal peripheral blood samples (*N* = 45) were collected from 18 patients of JAB-21822 cohort for CTCs analysis at baseline (BL, pre-treatment, *N* = 13), upon achieving partial response or stable disease (PR or SD, *N* = 24), and at confirmed disease progression (PD, *N* = 8) stage (Fig. 1B). Concurrently, serial computed tomography (CT) scans were acquired at standard efficacy assessment time points. Survival follow-up data, including PFS and OS events, are visually summarized for the cohort (Fig. 1C).

**Table 1:** Clinical characteristics of patients with KRAS^*G12C*^ mutation gastrointestinal tumor in the JAB-21822 cohort

Characteristics		ALL(*N* = 18)
Gender	Male	10 (55.6%)
	Female	8 (44.4%)
Ages(Years)	< 60	14 (77.8%)
	> = 60	4 (22.2%)
Tumor type	CRC	11 (61.1%)
	GC	2 (11.1%)
	PDAC	3 (16.7%)
	CCA	2 (11.1%)
MSI state	MSS/MSI-L	18 (100.0%)
	MSI-H	0 (0.0%)
ECOG at baseline	0	7 (38.9%)
	1	11 (61.1%)
Received radiotherapy	Yes	5 (27.8%)
	No	13 (72.25)
Liver metastasis	Yes	10 (55.6%)
	No	8 (44.4%)
Lung metastasis	Yes	8 (44.4%)
	No	10 (55.6%)
Peritoneal metastasis	Yes	10 (55.6%)
	No	8 (44.4%)
Ovary metastasis	Yes	2 (11.1%)
	No	16 (88.9%)
Treatment regimen	JAB-21822	9 (50.0%)
	JAB-21822 + CET	9 (50.0%)

*CRC* colorectal cancer, *GC* gastric cancer, *PDAC* pancreatic ductal adenocarcinoma, *CCA* cholangiocarcinoma, *MSS* microsatellite stability, *MSI-H* microsatellite instability-high, *MSI-L* microsatellite instability-low, *JAB-1822* glecirasib, *CET* cetuximab

### Glecirasib treatment induced dynamic changes in circulating tumor cell subtypes and their correlation with disease progression

**Figure. 2:**
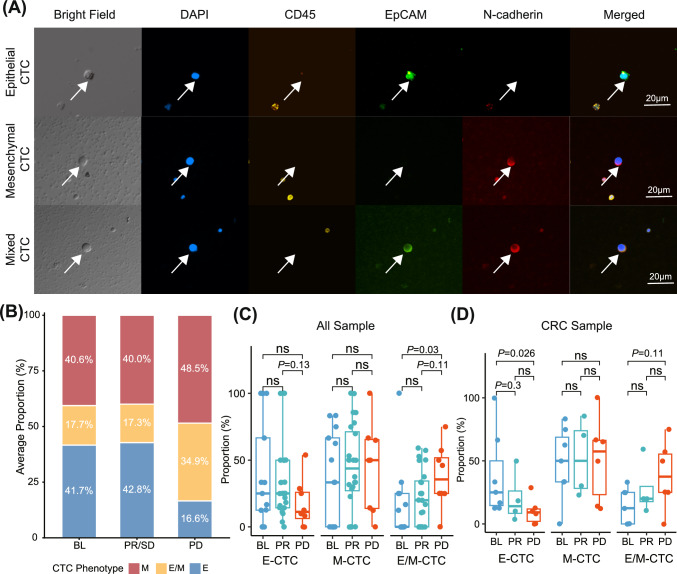
Dynamic evolution of CTC subtypes during *KRAS*^G*12*C^ targeted therapy. **A** Representative immunofluorescence images of the three CTC subtypes defined by EpCAM and N-cadherin expression: Epithelial (E-CTC; EpCAM^+^/N-cad^−^), Mesenchymal (M-CTC; EpCAM^−^/N-cad^+^), and Epithelial/Mesenchymal mixed (E/M-CTC; EpCAM^+^/N-cad^+^). Scale bar: 20 µm. **B** Stacked bar chart showing the proportional distribution of CTC subtypes at baseline (BL), on-treatment (PR/SD), and progressive disease (PD) timepoints. **C** Box plots comparing the proportion of E-CTCs, M-CTC and E/M-CTCs across treatment stages in the overall cohort (*n* = 18). **D** Subgroup analysis of CTC dynamics in colorectal cancer (CRC) patients (*n* = 11)

CTCs were isolated using a microfluidics platform (CTC100, Cellomics). Characterized using an integrated immunofluorescence assay, based on the expression of EpCAM (epithelial marker) and N-cadherin (mesenchymal marker), three CTCs subtypes were defined, DAPI^+^/CD45^−^/EpCAM^+^/N-cadherin^−^ E-CTCs, DAPI^+^/CD45^−^/EpCAM^−^/N-cadherin^+^ M-CTCs and DAPI^+^/CD45^−^/EpCAM^+^/N-cadherin^+^ E/M-CTCs (Fig. 2A). Longitudinal analysis of the proportional distribution of CTCs subtypes following glecirasib targeted therapy revealed a shift from a predominantly E-CTCs (41.7%) at baseline to M-CTCs (48.5%) at disease progression (PD). However, the proportion of E/M-CTCs significantly increased from 17.7% at baseline to 34.9% upon progression (Fig. 2B). The dynamic quantitative variation in each CTCs subtype following treatments showed a consistent trend of change. Compared to baseline, the proportion of E-CTCs showed a significant decrease at PD, particularly among colorectal cancer patients (*p* = 0.026). In contrast, the proportion of E/M-CTCs increased significantly (*p* = 0.030) (Fig. 2C-D). Taken together, these findings suggest that glecirasib targeted therapy exerts a selective pressure, driving dynamic repopulation of the CTCs pool and favoring clones with an E/M-CTCs phenotype upon the development of therapeutic resistance.

### Longitudinal CTCs evolution and correlation with radiographic assessment

**Fig. 3:**
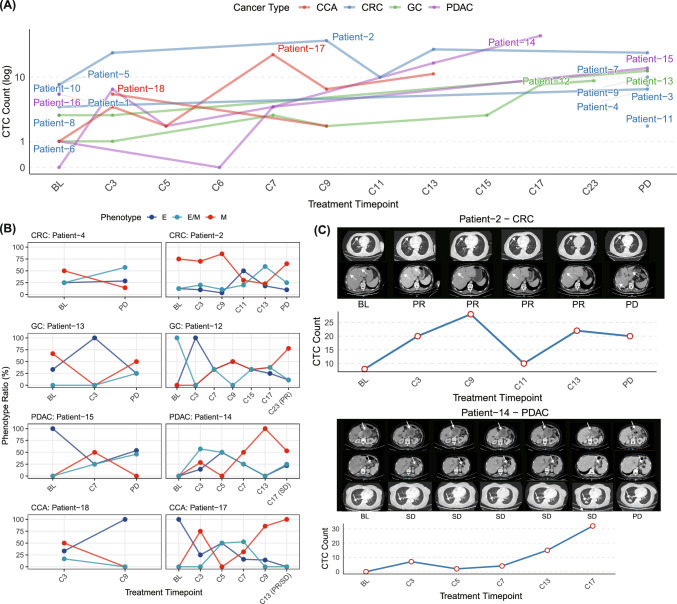
Longitudinal CTC evolution and correlation with radiographic assessment. **A** Spaghetti plot depicting changes in total CTC counts for each patient across successive timepoints. **B** Representative cases illustrating distinct CTC phenotype trajectories within rapid progressors (Patient-4, Patient-13, Patient-15, Patient-18) and long-term responders (Patient-2, Patient-12, Patient-14, Patient-17). **C** Integrated longitudinal analysis of CTC dynamics and radiographic assessment in two representative long-term responders (Patient-2 and Patient-14)

During prospective longitudinal surveillance follow-up, we observed that CTCs counts generally increased with tumor progression (Fig. 3A). Among patients treated with glecirasib targeted therapy across different cancer types, those with rapid disease progression (patient-4, patient-13, patient-15, patient-18) primarily exhibited dynamic changes in the E-CTCs subtype during treatment. In contrast, long-term responders (patient-2, patient-12, patient-14, patient-17) showed dynamics dominated by M-CTCs changes, with a decreasing trend in M-CTCs proportion during the treatment response phase and an increase upon disease progression (Fig. 3B). Furthermore, in long-term responders (e.g., Patient-2 and Patient-14), the inflection points of declining M-CTCs proportion aligned with clinical progression trend and were consistent with radiological efficacy assessments (Fig. 3C and Fig. S1). It is worth noting that in Patient-14, although the overall RECIST 1.1 based assessment of the three target lesions, indicated SD from C3 to C17, progressive enlargement of the pulmonary target lesion was observed beginning at C13, meeting the criteria for oligoprogression. Notably, the M-CTCs proportion reached an inflection point at C5 and began an upward trajectory from C7 onward, which not only correlated with the patient's clinical course but also preceded radiographic evidence of oligoprogression in the lung lesion (Fig. 3C). These findings indicate that phenotypic shifts within the CTCs compartment, particularly the expansion in total CTCs count and the M-CTCs subtype proportion, may function as an early cellular indicator of emerging treatment resistance, which may occur prior to the detection of anatomical changes by CT imaging.

### The presence of CTC subtypes at baseline is predictive of patient outcomes following glecirasib targeted therapy

**Fig. 4:**
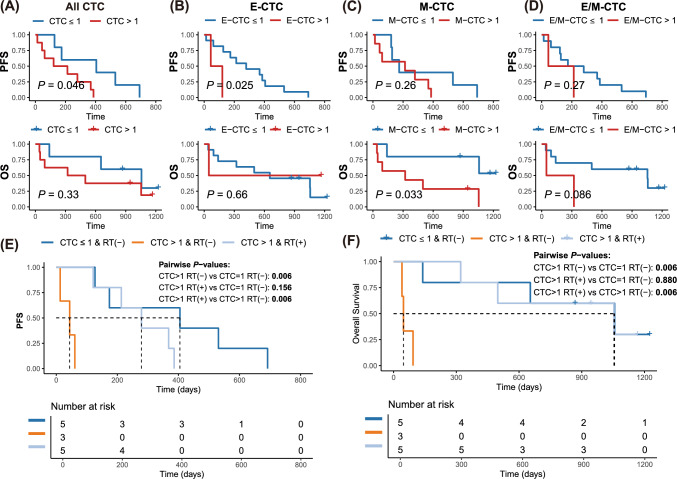
Prognostic associations of baseline CTC features and local radiotherapy. **A–D** Kaplan-Meier curves for progression-free survival (PFS) and overall survival (OS) in the overall cohort, stratified by baseline counts of total CTCs (**A**), E-CTCs (**B**), M-CTCs (**C**), and E/M-CTCs (**D**). **E–F** Kaplan-Meier analyses of PFS and OS stratified by the combination of baseline CTC burden and administration of local radiotherapy (RT). *P*-values indicate statistical significance determined by the log-rank test (*p* < 0.05)

Patients in the JAB-21822 cohort were stratified by baseline CTC count to assess its prognostic value. Based on the previous studies, a CTCs count of ≥ 3 per 7.5 mL of blood was widely regarded as a prognostic cutoff for metastatic CRC patients, showing significant association with survival outcomes such as PFS and OS across multiple platforms [17–19]. Accordingly, in the present study, we adopted a threshold of > 1 CTCs per 4 mL of blood for prognostic analysis. The results revealed that individuals with > 1 CTCs at baseline had a significantly shorter PFS than those with ≤ 1 CTCs (*p* = 0.046), while overall survival (OS) was not significantly different (Fig. 4A). Further analysis demonstrated that the E-CTCs ≤ 1 patient was significantly associated with longer PFS (*p* = 0.025; Fig. 4B). In addition, the M-CTCs ≤ 1 patient was showed longer OS (*p* = 0.033; Fig. 4C). Moreover, as compared to patients with higher E/M-CTCs levels, those with E/M-CTCs ≤ 1 exhibited a favorable trend toward improved overall survival (OS), though not statistically significant (*p* = 0.086) (Fig. 4D). Within the CRC subgroup has the same trend, and the OS of the E/M-CTCs ≤ 1 patient was increased significantly (*p* = 0.008; Fig. S2). In addition, Among the JAB-21822 cohort, five patients received local radiotherapy (RT) for progressive lesions after glecirasib-targeted therapy. In patients with baseline CTCs > 1, those who received RT had significantly longer PFS and OS than those not (Fig. 4E-F). Owing to the limitations of a small sample size, we hypothesize that combining KRAS^*G12C*^ targeted therapy with radiotherapy may improve patient outcomes.

## Discussion

CTCs are currently primarily classified into E-CTCs, M-CTCs, and E/M-CTCs. The dynamic shifts in CTCs subtype proportions are related to the reversible changes of epithelial-mesenchymal transition (EMT) and mesenchymal–epithelial transition (MET) in tumor cells [20]. The proportions changes of these CTC subtypes are closely associated with disease progression and can serve as important predictive biomarkers for tumor progression [11, 20]. Consequently, the CTC subtype composition exhibits dynamic changes at different stages of tumor development. Studies have shown that the EMT of CTCs in CRC was primarily regulated by the transcription factors such as SNAIL, SLUG, TWIST1/2, and ZEB1/2. These factors suppress E-cadherin expression and activate MMP and Vimentin, thereby promoting loss of cell adhesion and degradation of the extracellular matrix [20]**.** Moreover, in CRC, the M-CTCs subtype was significantly associated with early relapse, and the E/M-CTCs subtype exhibited greater survival and adhesive capabilities, promoting distant colonization and tumor progression [20]. In addition, the studies on breast cancer have also reported that CTCs undergoing EMT demonstrate increased metastatic potential and aggressiveness. M-CTCs serve as important biomarkers for disease progression [12], while E/M-CTCs subsets, with their enhanced metastatic and proliferative abilities, were more specifically associated with distant metastasis and poorer prognosis in breast cancer patients [21]. Our research also indicates that following the KRAS^*G12C*^ target therapy of glecirasib, the CTCs subtypes of the JAB-21822 cohort undergo dynamic evolution. The predominant subtype shifted from an E-CTCs dominant profile at baseline to an M-CTCs dominant profile at the time of disease progression, accompanied by a significant increase in the proportion of E/M-CTCs. Therefore, the increased proportion of M-CTCs and E/M-CTCs may be associated with resistance to glecirasib therapy.

CTCs are cancer cells shed from primary tumors or metastatic sites into the bloodstream, regarded as critical "seeds" in the metastatic cascade. In circulation, CTCs face severe survival challenges, including fluid shear stress, anoikis, and immune surveillance [22]. To survive under such extreme conditions and successfully colonize distant organs, CTCs have evolved multiple adaptive strategies, among which interactions with various cell types in peripheral blood play a key role [23]. For instance, interactions with platelets, neutrophils, macrophages, and cancer-associated fibroblasts (CAFs) can regulate EMT in CTCs, thereby promoting their survival, proliferation, metastasis, and therapy resistance [23]. Studies have shown that platelets can activate TGF-β/Smad, PI3K/Akt, and other signaling pathways in CTCs by secreting factors such as transforming growth factor-β (TGF-β) and platelet-derived growth factor (PDGF), thereby inducing EMT and endowing CTCs with a mesenchymal phenotype that enhances invasiveness and migratory ability [24, 25]. CTCs can also interact with neutrophils in various forms, including cell–cell adhesion, cytokine secretion, protease release, and neutrophil extracellular trap (NET) formation. These interactions can protect CTCs from immune clearance and facilitate metastasis. In the tumor microenvironment, neutrophils may release cytokines such as IL-6, potentially activating the STAT3 pathway in tumor cells to promote proliferation and drug resistance [26]. Tumor associated macrophages (TAMs) can induce EMT in CRC and promote the generation and metastasis of M-CTCs by modulating the STAT3/miR-506-3p/FoxQ1 axis [27]. CAFs secrete extracellular matrix proteins (e.g., fibronectin, collagen) and growth factors (e.g., HGF, IGF-1), activating EMT-related signaling pathways in CTCs and enhancing their invasive and migratory potential [28]. During the interaction between CTCs and other cells, reactivation of downstream signaling pathways such as RAF/MEK/ERK and PI3K/AKT/mTOR, as well as bypass activation of receptor tyrosine kinases (RTKs), can contribute to therapy resistance and the development of drug tolerance [8, 29, 30]. In-depth investigation into the interaction mechanisms between CTCs and peripheral blood components holds important implications for exploring strategies to overcome resistance to KRAS^*G12C*^ targeted therapy.

CTC detection holds significant clinical value in tumor progression and the indication of drug resistance. Dynamic monitoring of CTCs subtype composition can help predict the risk of recurrence in advance. CTC liquid biopsy has been approved by the FDA for monitoring disease progression and predicting poor prognosis in patients with metastatic colorectal cancer [20], breast cancer [12], and prostate cancer [15] et.al. Studies have shown that CTC subtype dynamics often precede radiological detection, several as an early warning indicator, and the changes in CTC count and subtype composition can predict tumor recurrence several months before it becomes visible on imaging. The research has found that in early-stage cancer cases, CTCs can be detected in peripheral blood even before any lesions visible on imaging [14, 31]. In lung adenocarcinoma (LUAD) patients who underwent curative resection, the median time from a positive CTC detection to radiological recurrence was 183 days, which was longer than the 70 days and 88 days median intervals reported in ctDNA-based studies. Notably, CTC detection signaled recurrence up to 354 days earlier than identification by CT scans [14]**.** Our study also found that during glecirasib treatment, patients with disease remission show a decrease in both CTC count and the proportion of M-CTCs, whereas those with disease progression exhibit the opposite trend. In the JAB-21822 cohort, patients presented with multiple target lesions. Although therapy response assessment according to RECIST 1.1 criteria indicated PR or SD, certain individual lesions exhibited progressive enlargement. For example, in patient-14, despite sustained SD based on overall evaluation, pulmonary metastases showed progressive growth beginning from cycle-7 (C7). The proportion of M-CTCs demonstrated a positive correlation with the progression of lung metastases, which were visible on imaging (CT). This result suggests that M-CTC levels may serve as a potential biomarker for tumor progression and treatment response prediction during glecirasib therapy.

The combination of targeted therapy and radiotherapy represents a key strategy for improving the prognosis of cancer patients with KRAS^*G12C*^ mutation [32–34]. Preclinical studies have demonstrated that KRAS^*G12C*^ inhibitors (adagrasib and sotorasib) can interfere with DNA damage repair pathways, thereby sensitizing tumor cells to radiotherapy-induced DNA double-strand breaks and functioning as "radiosensitizers" [33]. Meanwhile, radiotherapy can induce immunogenic cell death and release tumor neoantigens, but its efficacy is often limited by the immunosuppressive tumor microenvironment. KRAS^*G12C*^ inhibitors have been shown to downregulate immune checkpoint molecules (PD-1/PD-L1) on tumor cells, reduce immunosuppressive cells, M2-type macrophages and Tregs, and increase the infiltration of cytotoxic T cells, thereby amplifying the "in situ vaccine" effect of radiotherapy and potentially triggering systemic “abscopal effects” [35, 36]. During targeted therapy, patients often experience progression in a limited number of lesions (≤ 5), a scenario termed “oligoprogression”. Stereotactic body radiotherapy (SBRT) applied to such lesions can effectively eradicate acquired resistant clones, thereby extending the efficacy of the current targeted treatment regimen and delaying the need to switch systemic therapies [37]. KRAS^*G12C*^ mutated tumor was prone to multi-organ metastasis, highlighting the significant role of precise radiotherapy in oligometastatic disease management. The combination of KRAS^*G12C*^ inhibitors and radiotherapy has evolved from a theoretical concept into a highly promising clinical strategy. Notably, a retrospective study involving patients with KRAS^*G12C*^ mutation non-small cell lung cancer (NSCLC) has shown that thoracic radiotherapy combined with KRAS^*G12C*^ inhibitors achieves better local control rates without increasing toxicity [38]. Therefore, in CRC patients with KRAS^*G12C*^ mutation, the future integration of targeted therapy and radiotherapy may offer a new strategy for improving patient outcomes, warranting further in-depth investigation. Its value lies not only in synergistic efficacy during initial treatment but also in providing a potent localized approach for managing and even reversing resistance to targeted therapy.

This study also has several limitations. The relatively small sample size may affect the statistical power and the reliability of the conclusions. As a single-center study lacking multi-center validation, the generalizability of the findings may be restricted. In addition, factors such as tumor heterogeneity and individual patient differences were not fully accounted for in this study, which may influence the analysis and interpretation of the results. And, microfluidic technology has shown outstanding advantages in CTCs detection, such as high sensitivity and high‑purity capture. However, its limited ability to identify and capture circulating tumor microemboli (CTM). Most microfluidic designs rely on antibody‑based capture or physical properties to isolate individual CTCs. Although, due to CTMs larger size, complex structure, and potential masking of surface antigens, are prone to clogging, fragmentation, or loss in microchannels, resulting in lower capture efficiency. Moreover, many strategies focus on single CTC analysis and lack effective preservation and analysis of CTM specific biological features, such as intercellular junctions and heterogeneous composition. However, microfluidic technology demonstrates clear strengths in detecting DNA and RNA molecular signatures from CTCs. Based on integrated microfluidic chips capable of single‑cell capture, lysis, and nucleic acid extraction enable nucleic acid analysis from individual CTCs with minimal sample input, making them suitable for detecting rare mutations. Coupled with digital PCR, next‑generation sequencing (NGS), or electrochemical sensing, microfluidics can enable quantitative and rapid analysis of nucleic acid biomarkers from CTCs, offering dynamic insights into treatment response and resistance evolution. In conclusion, CTCs subtyping analysis provides a new perspective for tumor prognosis assessment of patients with KRAS^*G12C*^ mutation gastrointestinal tumors in the JAB-21822 cohort, and the combination of targeted therapy and radiotherapy offers an effective strategy for improving patient outcomes. However, these findings require further validation through large-scale, multi-center studies.

## Supplementary Information

Below is the link to the electronic supplementary material.

Supplementary file1 (DOCX 19 KB)

Supplementary file2 (PDF 1697 KB)

Supplementary file3 (PDF 103 KB)

Supplementary file4 (DOCX 17 KB)

## Data Availability

All other data are available in the article and its Supplementary files or from the corresponding author upon reasonable request. Source data are provided with this paper. Further information and reasonable requests for resources and reagents should be directed to and will be fulfilled by the lead contact, Jian Li (oncogene@163.com).
