# Correction: The dynamic evolution of circulating tumor cells during glecirasib treatment predicts survival and resistance in gastrointestinal tumors with KRAS^*G12C*^ mutation

**DOI:** 10.1007/s13577-026-01434-9

**Published:** 2026-08-12

**Authors:** Lei Jiang, Yiming Luo, Huan Liang, Xujiao Feng, Yan Huang, Yanyan Li, Zhenghang Wang, Ting Xu, Heng Zou, Zhi Li, Lin Shen, Yang Chen, Weihu Wang, Jian Li

**Affiliations:** 1 Department of Gastrointestinal Oncology, Key Laboratory of Carcinogenesis and Translational Research (Ministry of Education), Peking University Cancer Hospital and Institute, Beijing, China; 2 Department of Radiation Oncology, Key Laboratory of Carcinogenesis and Translational Research, Ministry of Education/Beijing), Peking University Cancer Hospital and Institute, Haidian District, Fucheng Road 52, Beijing, China; 3 Department of Medical Affairs, Cellomics (Shen Zhen) Limited, Shenzhen, China

**Correction: Human Cell (2026) 39:95** 10.1007/s13577-026-01405-0

In this article, the affiliation details for Lin Shen were incorrectly listed as “Department of Radiation Oncology, Key Laboratory of Carcinogenesis and Translational Research, Ministry of Education/Beijing), Peking University Cancer Hospital and Institute, Haidian District, Fucheng Road 52, Beijing, China” and “Department of Medical Affairs, Cellomics (Shen Zhen) Limited, Shenzhen, China”. These affiliations were included in error and should have been deleted.

