# Supplementary material for: The dynamic evolution of circulating tumor cells during glecirasib treatment predicts survival and resistance in gastrointestinal tumors with KRASG12C mutation
Source: Hum Cell. 2026 Jul 1;39(7):95. doi: 10.1007/s13577-026-01405-0 (PMC13323780; doi:10.1007/s13577-026-01405-0)
Supplement: Supplementary file 1 — Supplementary file1 (DOCX 19 KB) [file 13577_2026_1405_MOESM1_ESM.docx]

**Table S1**

**Multivariate Cox proportional hazards regression analysis of baseline total CTC count and clinical covariates for PFS and OS in the JAB-21822 cohort (*N*=18).**

| Model | Variable | HR | P-value |
| --- | --- | --- | --- |
| PFS | CTC-Group (＞1 vs ≤1) | 4.63 (1.03-30.27) | 0.046 |
|  | Gender (Male vs Female) | 0.54 (0.15-1.87) | 0.328 |
|  | Age | 0.99 (0.94-1.04) | 0.687 |
| OS | CTC-Group (＞1 vs ≤1) | 2.01 (0.51-9.14) | 0.319 |
|  | Gender (Male vs Female) | 1.46 (0.34-6.86) | 0.609 |
|  | Age | 1.03 (0.97-1.1) | 0.35 |

****Abbreviations:*** CTC, circulating tumor cell; HR, hazard ratio; PFS, progression-free survival; OS, overall survival.
