# Supplementary figures and images for: The dynamic evolution of circulating tumor cells during glecirasib treatment predicts survival and resistance in gastrointestinal tumors with KRASG12C mutation
Source: Hum Cell. 2026 Jul 1;39(7):95. doi: 10.1007/s13577-026-01405-0 (PMC13323780; doi:10.1007/s13577-026-01405-0)

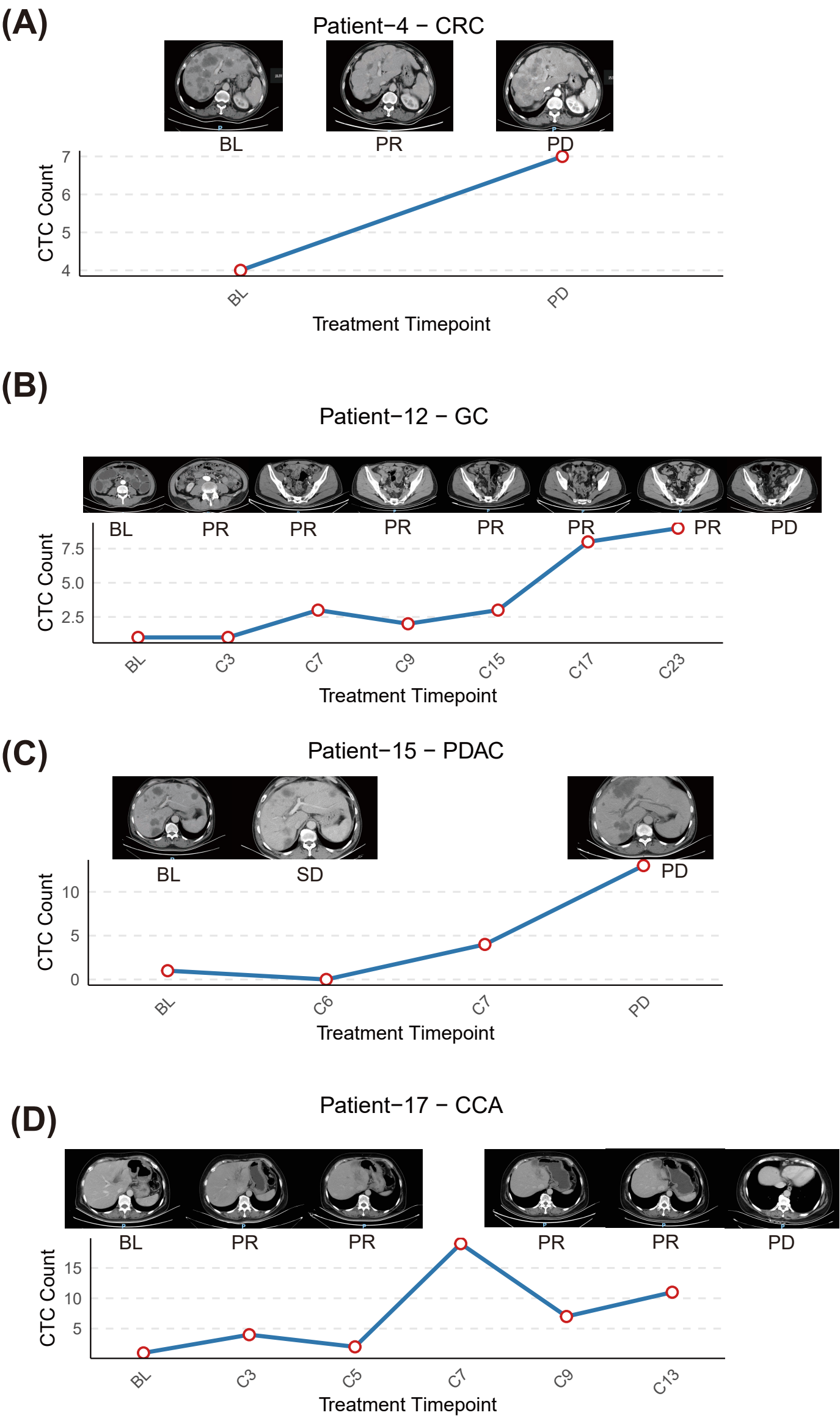

Supplement: Supplementary file 2 — Supplementary file2 (PDF 1697 KB) [file 13577_2026_1405_MOESM2_ESM.pdf]

## CRC Sample

(A)

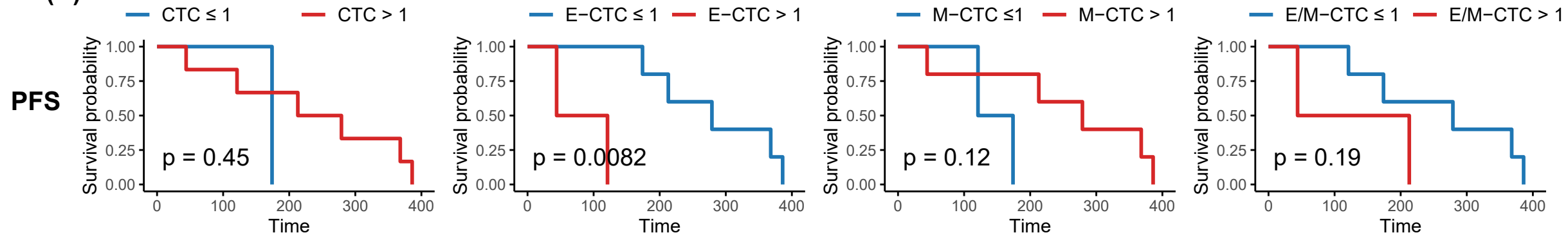

(B)

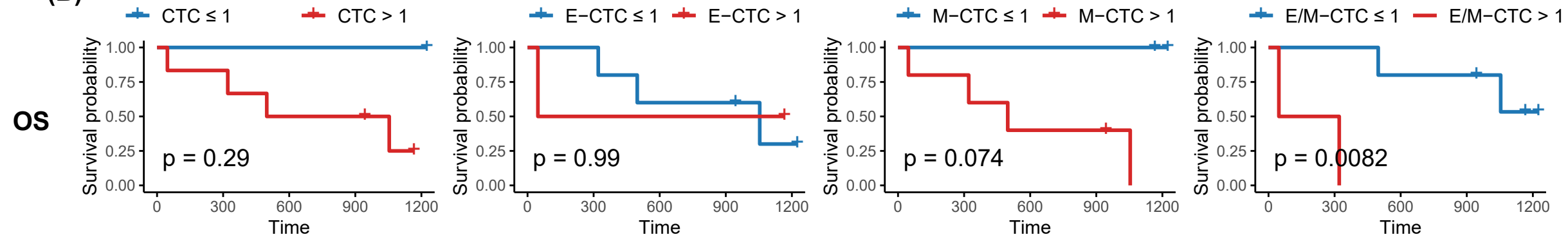

Supplement: Supplementary file 3 — Supplementary file3 (PDF 103 KB) [file 13577_2026_1405_MOESM3_ESM.pdf]
