# Supplementary material for: The dynamic evolution of circulating tumor cells during glecirasib treatment predicts survival and resistance in gastrointestinal tumors with KRASG12C mutation
Source: Hum Cell. 2026 Jul 1;39(7):95. doi: 10.1007/s13577-026-01405-0 (PMC13323780; doi:10.1007/s13577-026-01405-0)
Supplement: Supplementary file 4 — Supplementary file4 (DOCX 17 KB) [file 13577_2026_1405_MOESM4_ESM.docx]

**Supplementary figure legends**

**Supplementary Figure 1. Individual case studies of CTC dynamics and radiographic response.**
Integrated longitudinal analysis of CTC dynamics and radiographic assessment in four representative patients: (A) Patient-4 (Colorectal Cancer), (B) Patient-12 (Gastric Cancer), (C) Patient-15 (Pancreatic Ductal Adenocarcinoma), and (D) Patient-17 (Cholangiocarcinoma).

**Supplementary Figure 2. Prognostic significance of baseline CTC subtypes in the colorectal cancer subgroup.**

Kaplan-Meier analyses of progression-free survival (PFS) (A) and overall survival (OS) (B) in patients with CRC. Patients were stratified by baseline counts of total CTCs, E-CTCs, M-CTCs, and E/M-CTCs. *P*-values indicate statistical significance determined by the log-rank test (*p*＜0.05).
